# Effect of Traditional Cooking and In Vitro Gastrointestinal Digestion of the Ten Most Consumed Beans from the Fabaceae Family in Thailand on Their Phytochemicals, Antioxidant and Anti-Diabetic Potentials

**DOI:** 10.3390/plants11010067

**Published:** 2021-12-26

**Authors:** Duangjai Tungmunnithum, Samantha Drouet, Jose Manuel Lorenzo, Christophe Hano

**Affiliations:** 1 Department of Pharmaceutical Botany, Faculty of Pharmacy, Mahidol University, Bangkok 10400, Thailand; 2 Laboratoire de Biologie des Ligneux et des Grandes Cultures, INRAE USC1328, Campus Eure et Loir, Orleans University, 28000 Chartres, France; samantha.drouet@univ-orleans.fr; 3 Le Studium Institue for Advanced Studies, 1 Rue Dupanloup, 45000 Orleans, France; 4 Centro Tecnológico de la Carne de Galicia, Adva. Galicia n° 4, Parque Tecnológico de Galicia, San Cibrao das Viñas, 32900 Ourense, Spain; jmlorenzo@ceteca.net; 5 Área de Tecnología de los Alimentos, Facultad de Ciencias de Ourense, Universidad de Vigo, 32004 Ourense, Spain

**Keywords:** cooking effect, Fabaceae beans, phytochemical profiles, antioxidant effect, anti-diabetic effect, Thailand

## Abstract

The edible beans in Fabaceae have been used for foods and medicines since the ancient time, and being used more and more. It is also appeared as a major ingredient in dairy cooking menu in many regions including Thailand, a rich biodiversity country. Many studies reported on health benefits of their flavonoids, but there is no report on the effect of cooking on phytochemical profile and pharmacological potentials. Thus, this present study aims to complete this knowledge, with the 10 most consumed Fabaceae beans in Thailand, by determining the impact of traditional cooking and gastrointestinal digestion on their phytochemicals, their antioxidant and anti-diabetic activities using different in vitro and in cellulo yeast models. The results showed that *Vigna unguiculata* subsp. *sesquipedalis* were the richest source of phytochemicals, whereas the population of *V. mungo*, *Phaseolus vulgaris*, *V. angularis*, and *V. unguiculata* subsp. *sesquipedalis* were richest in monomeric anthocyanin contents (MAC). Furthermore, the results clearly demonstrated the impact of the plant matrix effect on the preservation of a specific class of phytochemicals. In particular, after cooking and in vitro digestion, total flavonoid contents (TFC) in *Glycine max* extract was higher than in the uncooked sample. This study is the first report on the influence of cooking and in vitro gastrointestinal digestion on the inhibition capacity toward advanced glycation end products (AGEs). All samples showed a significant capacity to stimulate glucose uptake in yeast model, and *V. angularis* showed the highest capacity. Interestingly, the increase in glucose uptake after in vitro digestion was higher than in uncooked samples for both *P. vulgaris* and *G. max* samples. The current study is the first attempt to investigate at the effects of both processes not only on the natural bioactive compounds but also on antioxidant and anti-diabetic activities of Thailand’s 10 most consumed beans that can be applied for agro-industrial and phytopharmaceutical sectors.

## 1. Introduction

The family Fabaceae (Leguminosae) is one of the most important economic vegetables that are consumed by people from every part of the world [1,2,3,4,5]. Fabaceae is a large plant family of terrestrial plants consisting of a large number of species (ca 19,400 species) [6]. Fabaceae plant have long been used as high nutrient food, cosmetics as well as medicines [4,5,7,8,9,10] The seed is the most useful part of Fabaceae species member, due to the fact that it contain high protein, low fat, low sugar as well as consisting of phytochemical compounds [2,3,4,5].Furthermore, there are many studies and reports from natural bioactive compounds and the related fields on health benefits of flavonoids and other phenolic compound from these edible seed species [2,4,5,8,9].

Thailand is one of the hot spots of biodiversity including the species richness and abundant of several plants. Edible Fabaceae species have long been used both foods and medicines since the ancient time, and currently being used more and more [11,12,13]. The most consumed beans species in Fabaceae family i.e., *Pisum sativum*, *Cajanus cajan*, *Vigna unguiculate*., *V*. *unguiculata* subsp. *sesquipedalis*, *V*. *radiata*, *V*. *mungo*, *V*. *angularis*, *Phaseolus vulgaris*, *Glycine max* and *Arachis hypogaea* are interested by many researchers to research on their natural product and biological activities [10,13,14], and these species usually use as the major ingredient in dairy cooking menu of Thai people. However, there is no report on the effect of cooking on phytochemical profile and their major pharmacological potentials i.e., antioxidant and anti-diabetic activities.

Legume seeds are rich in natural proteins, minerals, vitamins, and bioactive compounds, making them an important part of the human diet [15]. Bioactive phenolic compounds found in legume beans have been associated with a range of physiological and metabolic processes related to human health [16,17,18,19,20,21]. Phenolic acids, (iso)flavonoids, and anthocyanins are the main phenolic compounds present in legume beans [16,17]. Isoflavones are accumulated in relative high amount in legumes and their derived products. Isoflavones are phenolic compounds with a chemical structure similar to estradiol, and can mimic or inhibit the action or metabolism of this essential human hormone [17]. Genistein and daidzein are the most physiologically active isoflavones suitable for human health promotion [18,19,20,21].

Legume beans have been proposed as promising candidates for developing novel functional foods/vegetables because they are an important source of phytochemicals with high antioxidant potential, scavenging capacity, and the ability to interact and inhibit with some key proteins [22]. In addition to this strong antioxidant potential, legumes consumption has been inversely associated with type 2 diabetes incidence in adults [2,23]. Diabetes mellitus (DM) is on the rise at an alarming rate all over the world. DM is a severe, chronic, and complex metabolic syndrome with major consequences, such as long-term disruption, such as malfunction or failure of several vital organs [24]. The incidence of DM is steadily increasing and is estimated to exceed nearly 600 million by 2035 [25].

Type 2 diabetes (T2DM) is the most common type of diabetes and accounts for about 90% of all diabetes cases worldwide [25] that decrease the quality of life of those patients T2DM is characterized by hyperglycemia, impaired glucose tolerance, insulin resistance, and hyperlipidemia, and is caused by inefficient insulin utilization [24]. Postprandial hyperglycemia result in the glycation of both plasma and cellular proteins in DM patients, contributing to the development of diabetes complications. Thus, DM management necessitates precise postprandial glycemic control by lowering glucose absorption. This can be accomplished both by decreasing the activities of pancreatic α-amylase and intestinal α-glucosidase, which are essential enzymes involved in the digestion of the complex carbohydrates into absorbable monosaccharides from meals, or by affecting glucose facilitated diffusion through the cell [26]. Drugs such as acarbose, voglibose, and miglitol are clinically used for this purpose, but commonly cause significant gastrointestinal side effects such as stomach discomfort, gas, and diarrhea [27]. As a result, natural α-glucosidase and α-amylase inhibitors, sourced from our diet, have emerged as viable alternatives to supplement and/or possibly replace current medications [28,29,30,31]. Several natural plant compounds have emerged in recent decades as possible α-glucosidase and α-amylase inhibitors [28,29,30,31,32,33,34].

Traditionally the Fabaceae have been an important food daily consumed as fresh and cooked vegetables in Thailand. The incidence of DM is lower in Thailand (7.0%) than in western countries such as the United States (10.8%) or Germany (10.4%), but changes in dietary habits, particularly among young people, have favored the reported increase in recent years according to World Health Organization [25]. Legumes are considered a low glycemic index (GI) food, effective at reducing the postprandial glucose and insulin response compared to that of other carbohydrate-containing foods, such as rice or potatoes [2]. Several phytochemicals accumulated in their beans have been shown to inhibit α-glucosidase and α-amylase and/or to stimulate glucose uptake [35,36,37]. However, it is largely accepted that thermal processing during cooking, and digestion can greatly affect phytochemical concentration, it is therefore critical to consider these processes [38,39,40]. Many variables influence the effects of cooking and digestion, including the food matrix, cooking method/condition, and the phytochemical composition. To date, no study has considered the variability of phenolic compounds (phenolics, flavonoids, including the main bioactive isoflavones daidzein and genistein, and anthocyanins) from the most widely consumed beans in Thailand, and how traditional cooking and digestion affects the bio-accessibility as well as their antioxidant and anti-diabetic potentials.

The objective of this study is to complete this knowledge, with the 10 most consumed Fabaceae beans in Thailand, by determining the impact of traditional cooking and gastrointestinal digestion on their total content of phenolic, flavonoid (including HPLC determination of daidzein and genistein), and anthocyanin, as well as antioxidant and anti-diabetic activities determined using different in vitro and cellular assays.

## 2. Results and Discussion

### 2.1. Plant Collections

The most consumed beans from Fabaceae species throughout Thailand were collected from all floristic regions in Thailand (Table S1) with the collected locallities. According to the result from the field study and survey indicated P. vulgaris is the most common species in Thailand. The most abundant genus of these Fabaceae beans belongs to the genus Vigna which is its 4 species (*V. unguiculata, V. radiata, V. mungo* and *V. angularis*) distribute in every floristic region throughout Thailand (Table S1). 

### 2.2. Impact of Traditional Cooking and In Vitro Gastroinstetinal Digestion on Phytochemicals

**Figure 1:**
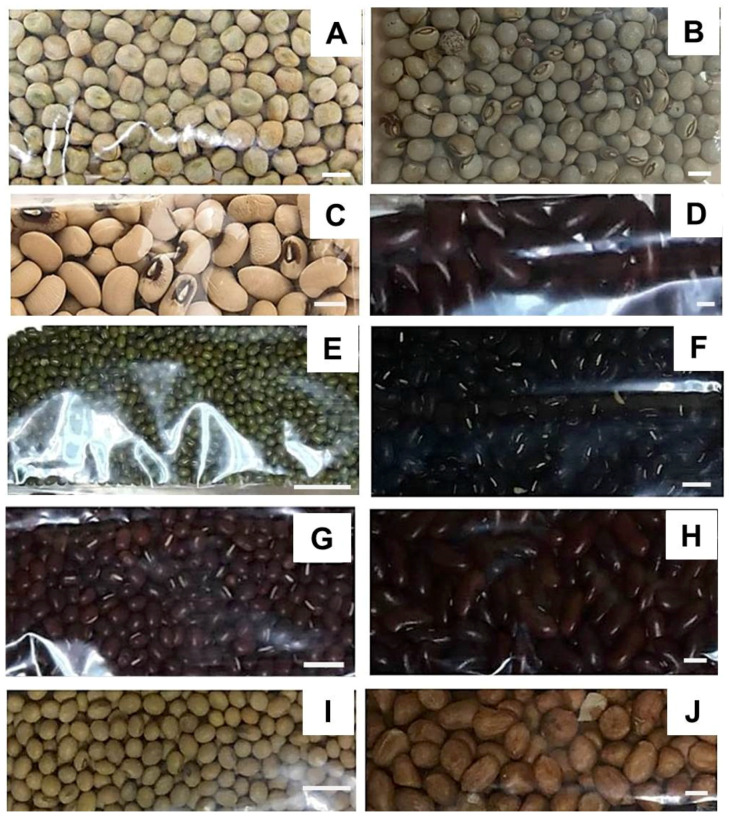
The collected seeds of Fabaceae plant in Thailand: (**A**) *P*. *sativum*, (**B**) *C*. *cajan*, (**C**) *V*. *unguiculate*., (**D**) *V*. *unguiculata* subsp. *sesquipedalis*, (**E**) *V*. *radiata*, (**F**) *V*. *mungo*, (**G**) *V*. *angularis*, (**H**) *P*. *vulgaris*, (**I**) *G*. *max*, (**J**) *A*. *hypogaea*; Bar scale = 1 cm. The photos were taken in Thailand by D.T.

Fabaceae beans are widely consumed and form an important part of the Thai diet [41]. Here, the ten most consummed Fabaceae beans (*P. sativum*, *C. cajan*, *G. max*, *P. vulgaris*, *V. angularis*, *V. mungo*, *V. radiata*, *V. unguiculata*, *V. unguiculata* subsp. *sesquipedalis*, and *A. hypogaea*) were studied (Figure 1).

The total phenolic (TPC), flavonoid (TFC), and monomeric anthocyanin (MAC) contents before (uncooked), as well as after traditional cooking (cooked) and in vitro gastro-intestinal digestion in these beans are reported in Table 1. The resulting estimated bio-accessibility after cooking and simulated digestion is shown in Table 2. In the present study, we focused on the impact of traditional cooking.

The phytochemical concentration and composition in beans differed widely across the diffent Fabaceae species (Table 1). *V. unguiculata* subsp. *sesquipedalis* beans were the richest source of phytochemicals, whereas *V. radiata* and *C. cajan* beans comparatively presented lower contents. Indeed, prior to cooking, the TPC ranged from 160.4 µg GAE/100 g DW (*V. radiata*) to 717.3 µg GAE/100 g DW (*V. unguiculata* subsp. *sesquipedalis*), the TFC ranged from 61.7 µg QAE/100 g DW (*C. cajan*) to 1510.7 µg QAE/100 g DW (*V. unguiculata* subsp. *sesquipedalis*), and the MAC ranged 3.4 µg CAE/100 g DW (*V. radiata*) to 41.6 µg CAE/100 g DW (*V. unguiculata* subsp. *sesquipedalis*).

The observed ranges of variation for the various species are consistent with published data [10,15,16,42,43]. The population *V. mungo*, *P. vulgaris*, *V. angularis*, and *V. unguiculata* subsp. *sesquipedalis* employed in this study were the richest sources of MAC (Table 1), which correlated well to their seedcoat color (Figure 1).

The cooking process lowered the availability of each phytochemical class (Table 2). Cooking had the least effect on TPC from *P. vulgaris*, whereas it had the highest effect on TFC from *V. unguiculata*. When compared to TPC and MAC, the TFC is the most affected by the cooking step, albeit this varies substantially from species to species. Simulated in vitro digestion, on the other hand, showed a mixed effect, with an overall negative influence on MAC, but increases in TPC and TFC for some species (*V. unguiculata*, *V. mungo*, *P. vulgaris*, *G. max*, or *V. unguiculata* subsp. *sesquipedalis*). Our results also clearly demonstrated the impact of the plant matrix effect on the preservation of a specific class of phytochemicals. In particular, after cooking and in vitro digestion, TFC in *G. max * extract was higher than in the corresponding uncooked sample.

**Figure 2:**
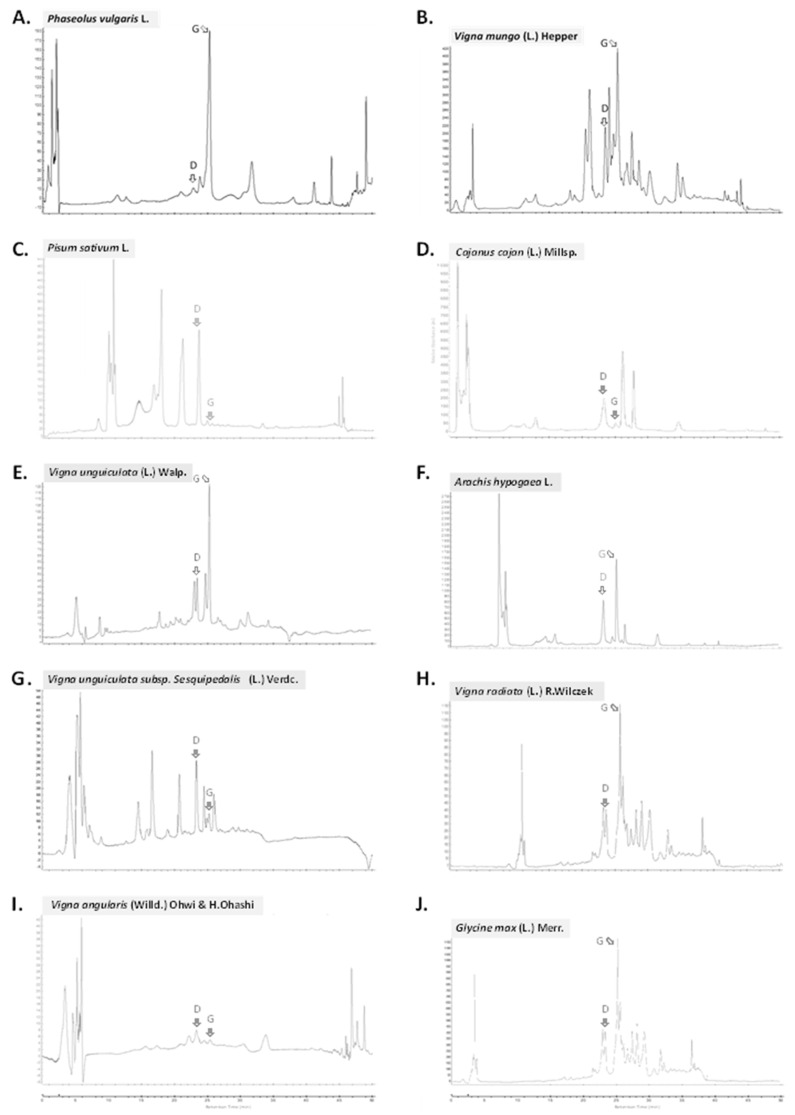
HPLC chromatograms (at 260 nm) of the 10 collected seeds of edible Fabaceae taxa in Thailand: (**A**) *P. vulgaris* from Chiang Mai; (**B**) *V. mungo* from Sukhothai; (**C**) *P. sativum* from Lampang; (**D**) *C. cajan* from Chumphon; (**E**) *V. unguiculata* from Chainat; (**F**) *A. hypogaea* from Surin; (**G**) *V. unguiculata* subsp. *sesquipedalis* from Chiang Mai; (**H**) *V. radiata* from Nakhon Sawan; (**I**) *V. angularis* from Phetchaburi; (**J**) *G. max* from Kalasin; D: daidzein; G: genistein.

The contents of the two main legume isoflavones, daidzein and genistein, were then determined by HPLC (Figure 2, Table 3).

Isoflavones are found in the highest concentration in Fabaceae beans and their derived products [17,44]. Genistein and daidzein are the most biologically active isoflavones that might benefit human health found in Fabaceae [18,19,20,21]. The variations in the contents for these two isoflavones were large, ranging from 6.5 µg/100 g DW (*V*. *angularis*) to 26,029.9 µg/100 g DW (*G*. *max*) for daidzein, and from 0.3 µg/100 g DW (*P*. *sativum*) to 82,514.7 µg/100 g DW (*G*. *max*) for genistein (Table 3). By far, *G*. *max* is the richest source of both isoflavones. These results are similar to those reported in the literature (i.e., ranging from 10,500 to 58,000 µg/100 g for Daidzein, and from 26,800 to 84,100 µg/100 g for genistein in soy bean for example) [17,44]. The complete ranges have been rewieved by [17,44]. Our results expand our understanding for Thai cultivars for which no database is currently available, as well as for species like *V*. *unguiculata* subsp. *sesquipedalis* that has never been reported previously.

The influence of cooking and in vitro digestion on these two isoflavones is shown in Table 4. 

Cooking reduces isoflavone levels by roughly 50%, similarly to TFC, however digestion has a less pronounced impact and even facilitates their releases (Table 4).

The phenolic content of vegetables, particularly flavonoids, has been recognized to be influenced by thermal processing [45], including cooking [38,46]. Many factors influence the effects of cooking, including the food matrix, cooking method/condition, and the chemical composition of the flavonoids [47]. The chemical nature of the flavonoids is another important factor that determines their fates during cooking. Similarly, digestion have been reported to greatly affect the bio-accessibility of various phenolic classes [40]. Moreover, several physiological factors such as pH, the presence of digestive enzymes, and bile salts have a substantial impact on the bio-accessibility of food components [39]. The low stability of anthocyanins during digestion have been previously reported [48]. On the contrary, some phenolic compounds in the seeds are not free but bound to the cell wall and/or complexed into macromolecular complex as observed in flax [45,49]. Thus in vitro digestion may result in their release, thus increasing their concentration, from the cell wall and/or macromolecular complex through the action of digestive enzymes such as α-amylase during the oral phase or hydrolosis as a consequence of the acidic pH during the stomach phase [49,50]. Additionally, some phytochemicals, such as isoflavones or hydroxycinnamic acids are stored as glycosides [17,49], and digestion may facilitate the release of aglycone forms, which might explain the rise found between the cooking and digestion phases. Consequently, all these observations might explain the increase in TFC and TPC observed for some of our Fabaceae samples.

### 2.3. Impact of Traditional Cooking and In Vitro Gastroinstetinal Digestion on In Vitro and Cellular Antioxidant Capacity

Fabaceae beans is a rich source of natural antioxidant [16]. The antioxidant capacity of a phenolic molecule is closely related to its chemical structure [51]. Cooking and/or digestion may alter the natural structure of these phytochemicals. Our next aim was to assess the influence of these activities on our collection of ten Fabaceae beans on their antioxidant capacity determined using three in vitro cell-free and one cellular assays (Table 5). 

Before cooking, the DPPH free radical scavenging activity of the extracts ranged from 36.1 µmol TE/g DW (*A*. *hypogaea*) to 84.1 µmol TE/g DW (*V. angularis*), the ABTS radical scavenging activity ranged from 52.6 µmol TE/g DW (*A. hypogaea*) to 114.5 µmol TE/g DW (*V. angularis*) µmol TE/g DW, and finally the FRAP reducing power ranged from 49.0 µmol TE/g DW (*C. cajan*) to 326.8 µmol TE/g DW (*V. unguiculata* subsp. *sesquipedalis*) (Table 5).

The results showed that cooking and in vitro digestion seriously negatively impact the in vitro antioxidant capacity. The digestive phase further decreased the free radical scavenging activity of the samples as observed for the DPPH and ABTS assays, whereas the reducing power, determined with the FRAP assay was less affected.

Because of the complex nature of phytochemicals, and in particular because antioxidant activity is strongly dependent on the reaction mechanism involved, the antioxidant activity of plant extracts cannot be measured using a single approach [52,53]. A variety of chemical or biological assays are required to evaluate the antioxidant activity and mode of action of a plant extract [52,53]. The in vitro cell-free antioxidant assays are based on different types of chemical reactions: the ABTS assay used a hydrogen atom transfer reaction (HAT), the FRAP assay used an electron transfer reaction (ET), and the DPPH assay used a mixture of both mechanisms [52,53,54,55].

Our results are consistent with previous research studies subjected to cooking or digestion, where significant decreases in antioxidant capacity were observed [38,39].

The validity of the in vitro cell-free antioxidant tests is restricted to the interpretation of the chemical reactivity. The validation using in vivo cellular antioxidant assay (CAA) is necessary. Here, the widely employed yeast cells have been used for CAA [56,57,58]. The results corroborated the trend shown in in vitro antioxidant assays at the cellular level with a reduction of the antioxidant potential after cooking and digestion. The differences observed in the impact of these two processes confirmed the influence of the matrix effect.

As a direct result of redox cellular imbalances, the generation of ROS and RNS rises with age, stress, or pollution, and has been connected to aging processes and perhaps contributing to the development of a range of degenerative diseases [59,60,61]. Yeast is a reliable eukaryotic model with well-known systems involved in oxidative stress defense and adaption that may readily be extended to humans [56,57]. Thus, the current findings backed up the probable preventive effect of Fabaceae phenolics against chronic degenerative diseases [62], and provided new information on the impact of cooking and in vitro digestion on Thailand’s ten most consumed beans, which could serve as a promising database for future epidemiological studies.

### 2.4. Impact of Traditional Cooking and In Vitro Gastroinstetinal Digestion on In Vitro and Cellular Anti-Diabetic Potential

Legumes consumption has been inversely associated with type 2 diabetes incidence in adults [2,23]. Pentosidine-like AGEs are mostly found in plasma and erythrocytes in humans, whereas vesperlysine-like AGEs are mostly found in diabetes patients’ lenses [63]. As a result, identifying particular inhibitors of various AGEs might be critical for targeting treatment action to a specific problem. Here, the impact of cooking and in vitro digestion was next evaluated on various potential anti-diabetic actions using in vitro and cellular assays (Table 6 and Table 7).

Fabaceae bean extracts showed significant inhibition of vesperlysine- and pentosidine-like advanced glycation end products (AGE) (Table 6). This inhibition was more pronounced for vesperlysine-like AGEs. A significant decrease of this capacity was observed with samples subjected to cooking. In vitro digestion had a less detrimental effect, and it even had a stimulating effect on *P. vulgaris* and *G. max* samples.

To the best of our knowledge, the present study is the first report on the influence of cooking and in vitro gastrointestinal digestion on the inhibition capacity toward vesperlysine- and pentosidine-like AGEs of different Fabaceae beans.

Several phytochemicals accumulated in Fabaceae beans have been already shown to inhibit α-glucosidase and α-amylase and/or to stimulate glucose uptake [35,36,37]. We next evaluated the influence of cooking and in vitro digestion on these actions for our Fabaceae collection (Table 7). 

The inhibition capacity toward both α-glucosidase and α-amylase enzymes of these bean extracts was here confirmed. The highest inhibitions were noted with uncooked beans of *V. unguiculata* subsp. *sesquipedalis* for both enzymes. Depending on the Fabaceae species studied, cooking and digestion lowered the inhibitory ability of practically all of the extracts with very distinct intensity, as for example the very significant reduction of the α-amylase inhibition of *V. mungo* sample *vs* almost no effect on the α-glucosidase and α-amylase inhibition capacities of *C. cajan* sample. On the opposite, the α-glucosidase inhibition capacity of *G. max* sample was stimulated by in vitro digestion. This could be linked to the capacity of in vitro digestion to stimulate the release of cell wall bound phenolics or aglycone observed for this sample (Table 1, Table 2, Table 3 and Table 4).

The control of hyperglycemia can be obtained using inhibitors of digestive enzymes α-glucosidase and/or α-amylase, but also by stimulating glucose uptake [35,36,37]. Postprandial hyperglycemia may result for example in the glycation of both plasma and cellular proteins in DM patients, contributing to the development of diabetes complications [24]. Additionally, here, all samples showed a significant capacity to stimulate glucose uptake, here determined using yeast model (Table 7). *V. angularis* showed the highest capacity to stimulate glucose uptake in this model. Compared to the other biological activities, the increase of glucose uptake, was less influenced by cooking and in vitro digestion. Interestingly, increase of glucose uptake after in vitro digestion was higher than in uncooked samples for both *P. vulgaris* and *G. max* samples.

Our results reveal that cooking and digestion had diverse potential antidiabetic actions in a species-dependent manner: acting as in vitro inhibitors of vesperlysine- and pentosidine-like AGEs, as well as of α-glucosidase and/or α-amylase enzymes, and by stimulating glucose uptake. We anticipate that these findings might be exploited in the future to correlate and/or explain findings from epidemiological studies.

### 2.5. Correlation Analysis

**Figure 3:**
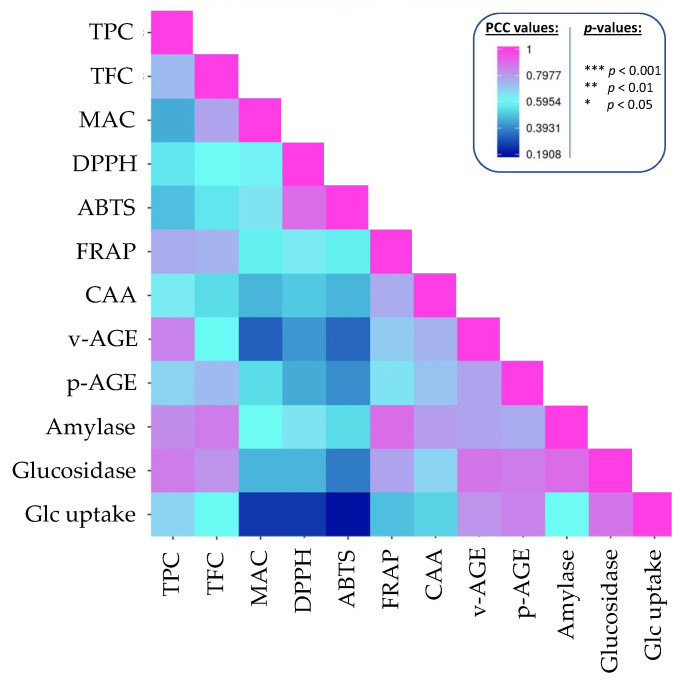
Correlogram analysis (Pearson coefficient correlation) between phytochemicals, antioxidant and anti-diabetic activities of extracts from beans from ten Fabaceae species subjected to traditional cooking and in vitro gastrointestinal digestion. The actual values and *p*-values are provided in Table S2.

Pearson correlation coefficient were calculated to establish possible correlations between a phytochemical class and one of the studied biological acitivities (Figure 3, Table S2).

TPC and TFC were significantly linked with all studied biological activities. In particular, high correlation were calculated (i) for TPC: with FRAP antioxidant capacity (PCC = 0.766), inhibition of vesperlysine-like AGEs (PCC = 0.847), α-glucosidase (PCC = 0.855) and α-amylase (PCC = 0.829) inhibition; (ii) for TFC: with with FRAP antioxidant capacity (PCC = 0.747), inhibition of pentosidine-like AGEs (PCC = 0.731), α-glucosidase (PCC = 0.863) and α-amylase (PCC = 0.808) inhibition.

On the contrary, inhibition of vesperlysine-like AGEs and increase of glucose uptake were not significantly linked to MAC. But MAC was significantly correlated with ABTS antioxidant capacity (PCC = 0.719).

There are no significant correlations with daidzein or genistein (*p* > 0.05). This does not necessarily imply that, contrary to what has been described in the literature, these two compounds cannot be responsible for all or part of these biological activities; rather, their range of variability in the 10 different species analyzed here is so wide that any correlations, if any, are disrupted. Furthermore, we have to keep in mind that this type of analysis generally does not consider the potential effects of interactions and/or synergies with other compounds or families of compounds.

## 3. Materials and Methods

### 3.1. Chemicals and Reagents

All the chemicals used in the present study are of analytical grade (Thermo Scientific, Illkirch, France). The reagents for all assays were from Merck (Saint-Quentin Fallavier, France) unless otherwise stated.

### 3.2. Plant Materials

The targeted taxa of Fabaceae species: *P. sativum*, *C. cajan*, *V. unguiculate.*, *V. unguiculata* subsp. *sesquipedalis*, *V. radiata*, *V. mungo*, *V. angularis*, *P. vulgaris*, *G. max*, *A. hypogaea* were studied. The collected plant materials were identified using the taxonomic key and description in the existing Floras [64,65,66], and compared with the herbarium specimens kept in the Prof. Kasin Suvatabandhu from Chulalongkorn University, (BCU) and the Forest Herbarium (BKF) which are the major national herbarium. The herbarium abbreviations are used according to Thiers [67]. After that the collected seeds were air-dried, as well as prepared following the World Health Organization [68] recommendations for this study.

### 3.3. Traditional Cooking

The collected seeds were divided into 2 groups per each population such as cooked and uncooked groups. For the cooked group, dry seeds were cooked following the traditional ways at the temperature 60 °C and 60 min following the previous investigation [14]. Part of the sample was then submitted to in vitro gastrointestinal digestion as described in the Section 3.4. Then, the extraction was processed as described in the Section 3.5 for both uncooked, cooked without in vitro gastrointestinal digestion and cooked with in vitro gastrointestinal digestion samples.

### 3.4. In Vitro Gastrointestinal Digestion

The assay was performed according to the procedures described by Mihaylova et al. [39] with minor modifications like the addition of a salivary phase as described by Aylanc et al. [69].

Briefly, simulated saliva fluid stock solution was combined with cooked Fabaceae beans (1.5 g/mL). This is followed by the addition of a 0.5 mL 500 U/mL α-amylase solution. The pH was adjusted to 7 with 1 mol/L NaOH, and the mixture was incubated in a water bath at 37 °C for 2 min with continual shaking in the dark.

The oral bolus was then combined with 1 mL of porcine pepsin stock solution (Sigma-Aldrich, Saint-Quentin Fallavier, France; 5520 U/mL made up in simulated gastric fluid electrolyte stock solution), 0.5 µL of 0.3 M CaCl_2_, and 50 µL of phospholipids for the stomach phase (0.17 mM in the final digestion mixture). The pH of the mixture was adjusted to 3.0 using 1 M HCl, and the volume of the liquid was increased to 5 mL using distilled water. The mixture was then incubated for 2 h at 37 °C in a shaking water bath with continual shaking. The pH was monitored on a regular basis and re-adjusted as needed with 1 M HCl.

Gastric chyme (5 mL) was combined with pancreatin solution (Sigma-Aldrich, Saint-Quentin Fallavier, France; 1.72 U/mL made up in simulated gastric fluid electrolyte stock solution based on trypsin activity,), 1.5 mL fresh bile extract (Sigma-Aldrich, Saint-Quentin Fallavier, France; 160 mM fresh bile salts in final mixture), 10 μL of 0.3 M CaCl_2_, 1 M NaOH to reach pH 7.5, and water to 10 mL total volume. After that, the mixture was incubated for 2 h at 37 °C in a shaking water bath. Throughout the procedure, the pH was tested on a regular basis and, if necessary, corrected with 1 M NaOH.

Instead of bean extract, water was utilized for the blank sample. For each analysis, the blank values were removed from the sample values. The digestion sample was centrifuged and kept at −20 °C before further analysis.

### 3.5. Extraction

Ultrasound-assisted extraction [70] was employed using an ultrasonic bath (USC1200TH, Prolabo, Sion, Switzerland) consisting of a 300 × 240 × 200 mm (inside dimension) tank with an electric power of 400 W equal to an acoustic power of 1 W/cm^2^ and a maximum heating power of 400 W. A frequency controller allowed to select the US frequency of the device, also equipped with a temperature regulator and an automatic digital timer. Using previously optimized extraction procedure, each sample (50 mg) was suspended in 10 mL 65% (*v*/*v*) aqueous ethanol and deposited in 50 mL quartz tubes with a vapor condenser and extracted during 40 min at an ultrasound frequency of 30 kHz. Following extraction, each extract was centrifuged for 15 min at 5000 g (Heraeus Biofuge Stratos, Thermo Scientific, Illkirch, France), and the supernatant was filtered using a syringe filter (0.45 m, Merck Millipore, Molsheim, France) before analysis. Each experiment was done in triplicate.

### 3.6. Determination of Total Phenolic Content (TPC)

The TPC was measured using the Folin–Ciocalteu protocol and microplate spectrophotometry as described previously [71]. Absorbance was measured at 725 nm with a spectrophotometer (BioTek ELX800 Absorbance Microplate Reader, BioTek Instruments, Colmar, France). A standard curve (0–40 µg/mL; R^2^ = 0.998) of gallic acid (Merck, Saint-Quentin Fallavier, France) was used to express the TPC in mg of gallic acid equivalents per g DW (mg GAE/g DW).

### 3.7. Determination of Total Flavonoid Content (TFC)

The colorimetric aluminum trichloride (AlCl_3_) method was used to determine TFC [72]. A 200 µL mixture was made in a microplate using 20 µL of extract, 10 µL of potassium acetate 1 M, 10 µL of AlCl_3_ (10% (*w*/*v*)), and 160 µL of deionized water. A microplate reader (Multiskan GO, Thermo Fischer Scientific, Illkirch, France) was used to measure the absorbance at 415 nm after 30 min of incubation at 25 °C in the dark. TFC was expressed in mg/g dry weight (DW) of quercetin equivalent using a five-point calibration line (linearity range from 0 to 40 g/mL quercetin concentration with an R^2^ of 0.998).

### 3.8. Determination of Monomeric Anthocyanin Content (MAC)

The colorimetric method was used to determine MAC [73]. Absorbance was measured at 510 and 700 nm with a spectrophotometer (BioTek ELX800 Absorbance Microplate Reader, BioTek Instruments, Colmar, France). A standard curve (0–100 µg/mL; R^2^ = 0.999) of cyanidin-3-*O*-glucoside (Merck, Saint-Quentin Fallavier, France) was used to express the TAC in mg of cyanidin-3-*O*-glucoside equivalents per g DW (mg GAE/g DW).

### 3.9. HPLC Analysis

Following extraction, each extract was centrifuged for 15 min at 5000 g (Heraeus Biofuge Stratos, Thermo Scientific, Illkirch, France), and the supernatant was filtered using a syringe filter (0.45 m, Merck Millipore, Molsheim, France) before analysis. HPLC was used to separate and identify the main isoflavonoids using a Varian system (Varian, Les Ulis, France) that included a Prostar 230 pump, Metachem Degasit, Prostar 410 autosampler, and Prostar 335 Photodiode Array Detector (PAD) and was controlled by Galaxie version 1.9.3.2 software (Varian, Les Ulis, France).

The separation was carried out on a Purospher RP-18 column (250 4.0 mm internal diameter; 5 m) (Merck Chemicals, Molsheim, France) at a temperature of 40 °C. The validated separation conditions were described previously [74]. The mobile phase was a mixture of water and phosphoric acid (1000:1, *v*/*v*) (solvent A), and water, acetonitrile and phosphoric acid (200:800:1, *v*/*v*/*v*) (solvent B). During the separation run (including 10 re-equilibration), the mobile phase composition varied according to a linear gradient as follows: B 0% (0 min) to 20% (5 min) to 100% (50 min) followed by 0% (60 min). Between each injection, a 10-min re-equilibration time was applied. The detection of compounds was set at 260 nm (corresponding to the λmax of the main compounds). Quantification was done based on assessment of retention times of commercial standard of daidzein and genistein (Merck, Saint-Quentin Fallavier, France).

### 3.10. In Vitro Cell Free Antioxidant Assays

The in vitro cell free DPPH (2,2-diphenyl-1-picrylhydrazyl), ABTS (2,2-azinobis(3-ethylbenzthiazoline-6-sulphonic acid) and FRAP (Ferric Reducing Antioxidant Power) assays were used to evaluate the in vitro cell free assays for determining antioxidant activity of the samples using the microplate-adapted protocols (Multiskan GO, Thermo Fischer Scientific, Illkirch, France) described by Drouet et al. [55] and Tungmunnithum et al. [71].

### 3.11. In Vitro Anti-Advanced Glycation End Products (AGE) Formation

The inhibitory capacity of advanced glycation end products (AGEs) formation was determined as described by Nazir et al. [53]. Extracts were prepared at a concentration of 50 μg/mL in DMSO (dimethyl sulfoxide) mixed with 20 mg/mL BSA (Sigma-Aldrich, Saint Quentin Fallavier, France). Both were prepared in phosphate buffer and 1 mL of 0.1 M phosphate buffer containing 0.02% (*w*/*v*) sodium azide at pH 7.4. The amount of fluorescent AGE formed was measured after incubation of mixture at 37 °C for 5 days in the dark by using a fluorescent spectrometer (Bio-Rad VersaFluor, Marne la Coquette, France) with an excitation wavelength set at 330 nm and emission wavelength set at 410 nm. For each extract, the percentage of anti-AGEs formation was showed as percentage inhibition relative to the corresponding control (addition of the same volume of DMSO).

### 3.12. In Vitro Anti-Diabetic Enzymes Inibition

#### 3.12.1. Pancreatic α-Amylase Inhibition

The inhibition of pancreatic α-amylase activity by Fabaceae bean extracts was studied according to the procedure described by Tiji et al. [75]. 

The assay mixtures contained 200 µL of pancreatic α-amylase enzyme solution (13 IU), 200 µL of phosphate buffer (0.02 M, pH = 6.9) and 200 µL of Fabaceae bean extracts at a 500 µg/mL or acarbose (positive standard drug control). The mixtures were pre-incubated at 37 °C for 10 min. Then, 200 µL of starch (1% (*w*/*v*)) dissolved in phosphate buffer was added to each tube and were incubated for 20 min at 37 °C. To stop the enzymatic reaction 600 µL of DNSA color reagent was added. Hereafter, the tubes were incubated for 8 min at 100 °C, before being put in an ice-cold-water bath for a few minutes. The mixture was diluted by adding 1 mL of distilled water and the absorbance was measured at 540 nm. Inhibition percentage was calculated using the formula bellows:

Inhibitory activity percentage = ((A_Control 540 nm_ − A_Test 540 nm_)/A_Control 540 nm_) × 100

With A_control 540 nm_: Absorbance of enzymatic activity without inhibitor, A_Test 540 nm_: Absorbance of enzymatic activity in the presence of Fabaceae bean extracts, or acarbose.

#### 3.12.2. Intestinal α-Glucosidase Inhibition

The effect of the Fabaceae bean extracts against intestinal α-glucosidase activity was quantified by monitoring the glucose release from sucrose degradation, according to the protocol described by Tiji et al. [75].

The assay mixtures contained 100 µL of sucrose (50 Mm), 1000 µL of phosphate-buffer (50 mM; pH = 7.5), and 100 µL of intestinal _-glucosidase enzyme solution (10 IU). Then, 10 µL of distilled water (control), acarbose (positive standard drug control), or Fabaceae bean extracts solutions at 500 µg/mL were added to the mixture. Then, tubes were incubated at 37 °C in a water bath for 25 min. The mixture was heated at 100 °C for 5 min to stop the enzymatic reaction, and the release of glucose was estimated by the glucose oxidase method using a commercially available auto-kit. The absorbance was measured at 500 nm, and the inhibition percentage was calculated using the below formula:

Inhibitory activity percentage = ((A_control 500 nm_ − A_Test 500 nm_)/A_control 500 nm_) × 100

With A_control 500 nm_: Absorbance of enzymatic activity without inhibitor, A_Test 500 nm_: Absorbance of enzymatic activity in the presence of Fabaceae bean extracts, or acarbose.

### 3.13. Yeast Culture Conditions

The yeast strain DBY746 (MAT leu2-3,112 his31 trp1-289a ura3-52 GAI+) culture was started with frozen stock plated onto an YPD medium (yeast extract peptone dextrose) (Sigma-Aldrich, Saint-Quentin Fallavier, France). Extract (CAJ-USE at a final concentration of 1 mg/mL) and resveratrol (RES, positive control, at a final concentration of 10 µM) were dissolved in cell culture grade dimethyl sulfoxide (DMSO; Sigma-Aldrich, Saint-Quentin Fallavier, France) and applied at a final DMSO concentration was 0.1% (*v*/*v*). Control yeast was inoculated with the same DMSO concentration. Survival was determined as previously described [58].

### 3.14. Cellular Antioxidant Assay

Yeast cells were first treated under the same conditions as mentioned above. Yeast cells were irradiated with a UV dose of 106.5 J/m2 UV-C (254 nm) under a Vilber VL-6.C filtered lamp (Thermo Fisher Scientific, Villebon-sur-Yvette, France), and incubated at 28 °C with orbital shaking at 120 rpm in the dark in complete 2.0% (*w*/*v*) glucose YPD medium (Sigma Aldrich, Saint-Quentin Fallavier, France) as previously described [58]. The same conditions were used to grow non-irradiated cells. Hour 0 of the oxidative stress experiment was considered irradiation. 

The dihydrorhodamine-123 (DHR-123) fluorescent dye (Sigma-Aldrich, Saint-Quentin Fallavier, France) was used to assess the quantity of reactive oxygen and nitrogen species. Approximately 10^8^ yeast cells were washed twice in PBS, resuspended in PBS containing 0.4 M DHR-123, and incubated for 10 min in the dark at 28 °C in the presence of extract, RES or DMSO (control cells). The fluorescence signal (ex = 505 nm, em = 535 nm) was measured using the VersaFluor Fluorimeter after two washes with PBS (Biorad, Marnes-la-Coquette, France).

### 3.15. Glucose Uptake Assay in Yeast Cell

The procedure described by Khan et al. [26]. The yeast culture was centrifuged for 5 min at 3000 g. The yeast cell pellet was collected for the experiment and the supernatant was discarded. In addition, a 10% (*v*/*v*) yeast suspension was made in sterilized deionized water. Fabaceae bean extract (500 µg/mL) was added separately to 1.0 mL of glucose solution (25 mM) and incubated for 10 min at 37 °C. In each extract-glucose solution, glucose uptake was started by adding 100 µL of yeast suspension. The suspension of the combination was vortexed and then incubated at 37 °C for 60 min. The culture was centrifuged (3000 g, 5 min) after 60 min of incubation, and the supernatant was used to measure the quantity of glucose present.

The percentage increase of glucose uptake in yeast cells was calculated using the equation:

Increase in uptake (%) = (absorbance of sample − absorbance of control) × 100/absorbance of sample

With absorbance of control reaction containing all reagents excluding the test sample; absorption of sample reaction containing Fabaceae bean extract.

### 3.16. Bio-Accessibility of Phytochemicals

Bio-accessibility (%) was defined as the content of the compound released in the cooking and simulated digestion processes compared to the content of the compound in the sample, and the value was calculated according to the equation [39]:

Bio-accessibility (%) = (C_f_/C_0_) × 100

where C_f_ is the final concentration of the compound (released during cooking or simulated digestion) or activity and C_0_ is the initial concentration of the same compound or activity.

### 3.17. Statistical Analysis

Statistical analyses were performed with XLSTAT 2019 suite (Addinsoft, Paris, France). Data composed of at least three independent replicates were presented using the means and standard deviations. Student *t*-test was carried out for statistical comparative analysis. Significant thresholds at *p* < 0.05, 0.01 and 0.001 were represented by *, ** and ***, respectively. Different letters were used to indicate significant thresholds at *p* < 0.05.

## 4. Conclusions

To understand the effects of cooking and digesting on phytochemical and biological activity is critical and useful for agro-industrial and phytopharmaceutical sectors. The majority of the previous research focused on the impact of cooking or digesting on a particular species or the cultivars of the same vegetable species. This current study is the first report to take a look at the effects of both processes on the phytochemical profiles as well as on the antioxidant and anti-diabetic activities of Thailand’s ten most consummed beans.The discovery of this study is the frontier knowledge to step further to animal and clinical studies using this Fabaceae beans as the potential raw plant material for health beneficial effects.

## Figures and Tables

**Table 1:** Evolution of total phenolic content (TPC), total flavonoid content (TFC) and monomeric anthocyanin content (MAC) before (uncooked), and after traditional cooking (cooked) and after traditional cooking followed by in vitro gastro-intestinal digestion (in vitro Digestion) from beans from ten Fabaceae species.

Plant Species	TPC (mg GAE/100 g DW)			TFC (mg QAE/100 g DW)			MAC (mg CAE/100 g DW)		
	Uncooked	Cooked	In Vitro Digestion	Uncooked	Cooked	In Vitro Digestion	Uncooked	Cooked	In Vitro Digestion
*P*. *sativum*	558.2 ± 44.3 ^a^	520.5 ± 67.3 ^ab^	452.5 ± 8.7 ^b^	348.3 ± 24.2 ^a^	210.1 ± 26.1 ^b^	246.9 ± 36.2 ^b^	8.8 ± 2.0 ^a^	4.0 ± 2.7 ^ab^	1.7 ± 1.0 ^b^
*C*. *cajan*	201.4 ± 23.6 ^a^	172.4 ± 9.6 ^a^	187.6 ± 8.5 ^a^	61.7 ± 2.3 ^a^	46.5 ± 8.4 ^a^	52.7 ± 14.8 ^a^	5.3 ± 0.5 ^a^	2.2 ± 1.6 ^b^	2.8 ± 1.5 ^ab^
*V*. *unguiculata*	337.6 ± 14.6 ^a^	120.1 ± 20.5 ^c^	200.7± 12.1 ^b^	444.5 ± 16.8 ^a^	78.8 ± 13.5 ^c^	175.3 ± 9.8 ^b^	19.0 ± 1.5 ^a^	16.1 ± 3.9 ^a^	3.8 ± 1.3 ^b^
*V*. *mungo*	547.2 ± 14.1 ^a^	158.8 ± 77.8 ^c^	460.9 ± 5.6 ^b^	872.1 ± 25.7 ^a^	211.5 ± 23.4 ^c^	525.5 ± 27.8 ^b^	27.9 ± 1.2 ^a^	14.9 ± 4.8 ^b^	5.8 ± 2.6 ^c^
*P*. *vulgaris*	440.2 ± 12.7 ^b^	184.3 ± 31.5 ^c^	496.0 ± 8.5 ^a^	355.2 ± 24.4 ^a^	161.6 ± 25.1 ^c^	294.1 ± 20.0 ^b^	17.3 ± 2.8 ^a^	9.7 ± 3.6 ^b^	1.1 ± 0.4 ^c^
*G*. *max*	483.4 ± 13.3 ^a^	268.1 ± 86.7 ^b^	480.3 ± 14.9 ^a^	235.6 ± 22.0 ^b^	105.6 ± 24.4 ^c^	386.7 ± 18.9 ^a^	11.6 ± 1.0 ^a^	5.2 ± 1.8 ^b^	1.9 ± 0.6 ^c^
*V*. *angularis*	697.7 ± 36.7 ^a^	415.0 ± 17.9 ^c^	500.7 ± 21.9 ^b^	1076.2 ± 38.9 ^a^	456.8 ± 39.1 ^b^	475.3 ± 29.8 ^b^	21.2 ± 0.4 ^a^	18.7 ± 1.6 ^b^	8.8 ± 2.3 ^c^
*A*. *hypogaea*	670.8 ± 6.2 ^a^	184.4 ± 80.1 ^c^	422.0 ± 15.1 ^b^	161.3 ± 12.5 ^a^	126.2 ± 16.6 ^a^	132.7 ± 18.3 ^a^	7.0 ± 0.5 ^a^	4.3 ± 1.9 ^ab^	1.9 ± 0.3 ^b^
*V*. *radiata*	160.4 ± 12.3 ^a^	107.8 ± 23.4 ^b^	120.5 ± 8.0 ^b^	66.5 ± 3.1 ^a^	46.2 ± 7.4 ^b^	43.2 ± 7.1 ^b^	3.4 ± 0.9 ^a^	1.9 ± 0.4 ^b^	0.4 ± 0.1 ^c^
*V*. *unguiculata* subsp. *sesquipedalis*	717.3 ± 37.1 ^a^	511.6 ± 16.5 ^c^	576.9 ± 13.5 ^b^	1510.7 ± 76.1 ^a^	396.0 ± 20.7 ^c^	885.0 ± 22.4 ^b^	41.6 ± 7.2 ^a^	23.7 ± 3.9 ^b^	10.4 ± 1.8 ^c^

Different superscript letters indicate significant differences at *p* < 0.05.

**Table 2:** Bio-accessibility (in %) of total phenolic content (TPC), total flavonoid content (TFC) and monomeric anthocyanin content (MAC) after traditional cooking (cooked) and after traditional cooking followed by in vitro gastro-intestinal digestion (Digestion) from beans from ten Fabaceae species.

Plant Species	TPC		TFC		MAC	
	Cooking	Digestion	Cooking	Digestion	Cooking	Digestion
*P*. *sativum*	93.2%	81.1%	60.3%	70.9%	45.7%	19.4%
*C*. *cajan*	85.6%	93.1%	75.5%	85.5%	43.2%	54.2%
*V*. *unguiculata*	35.6%	59.5%	17.7%	39.5%	84.8%	20.2%
*V*. *mungo*	29.0%	84.2%	24.3%	60.3%	53.6%	21.0%
*P*. *vulgaris*	41.9%	112.7%	45.5%	82.8%	56.2%	6.6%
*G*. *max*	55.5%	99.4%	44.8%	164.2%	45.5%	17.0%
*V*. *angularis*	59.5%	71.8%	42.4%	44.2%	88.4%	41.6%
*A*. *hypogaea*	27.5%	62.9%	78.3%	82.3%	63.2%	27.5%
*V*. *radiata*	67.2%	75.2%	69.5%	65.1%	55.9%	13.1%
*V*. *unguiculata* subsp. *sesquipedalis*	71.3%	80.4%	26.2%	58.6%	57.1%	25.0%

**Table 3:** Evolution of daidzein and genistein contents before (uncooked), and after traditional cooking (cooked) and after traditional cooking followed by in vitro gastro-intestinal digestion (in vitro Digestion) from beans from ten Fabaceae species.

Plant Species	Dadzein (µg/100 g DW)			Genistein (µg/100 g DW)		
	Uncooked	Cooked	In vitro Digestion	Uncooked	Cooked	In Vitro Digestion
*P*. *sativum*	28.6 ± 1.2 ^a^	13.3 ± 2.1 ^b^	24.6 ± 2.8 ^a^	0.3 ± 0.8 ^a^	nd ^b^	0.3 ± 0.1 ^a^
*C*. *cajan*	13.5 ± 0.3 ^a^	6.7 ± 1.7 ^b^	13.0 ± 1.8 ^a^	1.2 ± 0.3 ^a^	0.9 ± 0.3 ^a^	0.8 ± 0.3 ^a^
*V*. *unguiculata*	22.9 ± 1.5 ^a^	14.5 ± 2.4 ^b^	20.1 ± 3.2 ^a^	8.1 ± 0.5 ^a^	6.4 ± 1.1 ^a^	7.8 ± 1.6 ^a^
*V*. *mungo*	31.2 ± 2.9 ^a^	14.5 ± 2.1 ^b^	26.5 ± 2.1 ^a^	53.2 ± 0.8 ^a^	27.4 ± 2.1 ^c^	44.8 ± 1.7 ^b^
*P*. *vulgaris*	7.3 ± 1.2 ^a^	3.5 ± 2.2 ^b^	6.8 ± 0.9 ^ab^	129.2 ± 6.2 ^a^	68.1 ± 4.5 ^c^	98.3 ± 6.1 ^b^
*G*. *max*	26,029.9 ± 233.3 ^a^	11,527.2 ± 312.1 ^c^	24,136.7 ± 214.5 ^b^	82,514.7 ± 267.3 ^a^	39,843.4 ± 217.8 ^c^	68,175.3 ± 325.4 ^b^
*V*. *angularis*	6.5 ± 0.6 ^a^	2.5 ± 1.8 ^b^	5.7 ± 1.7 ^ab^	2.5 ± 1.0 ^a^	1.1 ± 1.0 ^a^	2.0 ± 1.1 ^a^
*A*. *hypogaea*	56.7 ± 6.1 ^a^	24.3 ± 11.2 ^b^	48.5 ± 12.3 ^ab^	80.7 ± 0.9 ^a^	18.6 ± 3.3 ^b^	72.3 ± 11.5 ^a^
*V*. *radiata*	49.1 ± 3.0 ^a^	27.8 ± 9.3 ^b^	42.5 ± 7.3 ^ab^	298.2 ± 2.4 ^a^	156.2 ± 16.5 ^c^	221.0 ± 26.7 ^b^
*V*. *unguiculata* subsp. *sesquipedalis*	54.3 ± 5.1 ^a^	12.1 ± 3.6 ^b^	45.7 ± 2.8 ^a^	16.7 ± 1.0 ^a^	7.6 ± 3.1 ^b^	9.7 ± 1.9 ^b^

nd: not detected; Different superscript letters indicate significant differences at *p* < 0.05.

**Table 4:** Bio-accessibility (in %) of daidzein and genistein after traditional cooking (cooked) and after traditional cooking followed by in vitro gastro-intestinal digestion (Digestion) from beans from ten Fabaceae species.

Plant Species	Daidzein		Genistein	
	Cooking	Digestion	Cooking	Digestion
*P*. *sativum*	46.5%	86.2%	-	106.9%
*C*. *cajan*	50.0%	96.6%	82.1%	69.2%
*V*. *unguiculata*	63.5%	87.9%	78.6%	97.0%
*V*. *mungo*	46.5%	85.0%	51.5%	84.3%
*P*. *vulgaris*	48.4%	94.8%	52.7%	76.1%
*G*. *max*	44.3%	92.7%	48.3%	82.6%
*V*. *angularis*	39.6%	88.6%	43.9%	79.4%
*A*. *hypogaea*	42.9%	85.6%	23.1%	89.5%
*V*. *radiata*	56.8%	86.6%	52.4%	74.1%
*V*. *unguiculata* subsp. *sesquipedalis*	22.4%	84.3%	45.6%	58.1%

**Table 5:** Evolution of in vitro cell-free (DPPH, ABTS and FRAP) and cellular (CAA) antioxidant activity before (uncooked), and after traditional cooking (cooked) and after traditional cooking followed by in vitro gastro-intestinal digestion (in vitro Digestion) from beans from ten Fabaceae species.

Plant Species	DPPH (µmol TE/g DW)			ABTS (µmol TE/g DW)			FRAP (µmol TE/g DW)			CAA (% ROS/RNS Inhibition)		
	Uncooked	Cooked	In Vitro Digestion	Uncooked	Cooked	In Vitro Digestion	Uncooked	Cooked	In Vitro Digestion	Uncooked	Cooked	In Vitro Digestion
*P. sativum*	69.8 ± 1.9 ^a^	54.7 ± 1.8 ^b^	42.2 ± 8.5 ^b^	61.6 ± 3.8 ^b^	89.2 ± 2.7 ^a^	48.9 ± 1.0 ^c^	243.3 ± 8.0 ^a^	96.5 ± 7.4 ^c^	182.8 ± 15.0 ^b^	83.2 ± 8.8 ^a^	47.8 ± 4.9 ^c^	57.2 ± 1.4 ^b^
*C. cajan*	47.2 ± 0.4 ^a^	42.1 ± 3.7 ^a^	34.0 ± 1.8 ^b^	66.9 ± 0.8 ^a^	46.1 ± 1.6 ^b^	41.4 ± 1.5 ^b^	49.0 ± 6.9 ^a^	24.9 ± 8.1 ^b^	51.3 ± 2.7 ^a^	61.3 ± 3.8 ^a^	37.3 ± 4.6 ^b^	42.8 ± 3.2 ^b^
*V. unguiculata*	38.9 ± 0.1 ^a^	32.3 ± 2.3 ^b^	24.9 ± 4.3 ^b^	56.2 ± 0.1 ^a^	39.1 ± 3.9 ^b^	22.4 ± 1.3 ^c^	173.4 ± 3.5 ^a^	54.7 ± 4.2 ^b^	58.9 ± 9.2 ^b^	76.5 ± 1.5 ^a^	48.5 ± 3.6 ^b^	57.2 ± 6.1 ^b^
*V. mungo*	56.4 ± 1.5 ^a^	44.7 ± 2.1 ^b^	33.3 ± 1.3 ^c^	78.7 ± 3.0 ^a^	52.1 ± 4.8 ^b^	28.9 ± 2.6 ^c^	296.7 ± 4.7 ^a^	78.9 ± 5.9 ^c^	155.2 ± 7.8 ^b^	85.4 ± 6.4 ^a^	33.3 ± 1.3 ^c^	49.4 ± 8.5 ^b^
*P. vulgaris*	51.8 ± 1.0 ^a^	45.9 ± 2.3 ^b^	31.5 ± 1.8 ^c^	72.8 ± 2.1 ^a^	53.7 ± 3.6 ^b^	29.5 ± 0.4 ^c^	179.6 ± 3.8 ^a^	64.2 ± 8.7 ^b^	56.3 ± 5.4 ^b^	79.2 ± 4.6 ^a^	41.0 ± 8.1 ^b^	52.2 ± 4.9 ^b^
*G. max*	48.1 ± 1.3 ^a^	36.2 ± 4.3 ^b^	29.8 ± 3.7 ^b^	68.1 ± 2.6 ^a^	44.0 ± 1.8 ^b^	33.1 ± 0.6 ^c^	145.5 ± 5.2 ^a^	70.5 ± 8.3 ^c^	114.5 ± 14.3 ^b^	75.9 ± 5.9 ^a^	42.8 ± 2.4 ^c^	58.0 ± 5.2 ^b^
*V. angularis*	84.1 ± 0.8 ^a^	62.1 ± 2.9 ^b^	66.3 ± 6.8 ^b^	114.5 ± 1.6 ^a^	98.7 ± 1.6 ^b^	71.5 ± 2.3 ^c^	320.0 ± 7.2 ^a^	130.7 ± 16.6 ^c^	280.5 ± 12.3 ^b^	87.8 ± 5.0 ^a^	48.9 ± 9.6 ^b^	55.6 ± 2.4 ^b^
*A. hypogaea*	36.1 ± 1.1 ^a^	26.4 ± 3.6 ^b^	30.6 ± 4.2 ^ab^	52.6 ± 2.2 ^a^	44.3 ± 1.9 ^b^	45.5 ± 0.3 ^b^	294.1 ± 4.8 ^a^	118.5 ± 6.2 ^b^	69.0 ± 9.2 ^c^	83.5 ± 5.2 ^a^	35.2 ± 3.4 ^c^	62.2 ± 3.5 ^b^
*V. radiata*	46.5 ± 1.4 ^a^	33.1 ± 4.9 ^b^	24.4 ± 4.8 ^b^	66.0 ± 2.9 ^a^	50.1 ± 0.4 ^b^	33.3 ± 0.1 ^c^	144.4 ± 4.3 ^a^	59.9 ± 9.1 ^c^	100.6 ± 10.7 ^b^	75.4 ± 6.1 ^a^	41.5 ± 7.3 ^b^	50.7 ± 11.4 ^ab^
*V. unguiculata* subsp. *sesquipedalis*	72.7 ± 1.1 ^a^	52.0 ± 5.5 ^b^	30.9 ± 4.7 ^c^	99.7 ± 2.2 ^a^	66.7 ± 3.9 ^b^	39.9 ± 1.8 ^c^	326.8 ± 3.2 ^a^	97.1 ± 2.8 ^c^	187.2 ± 13.8 ^b^	87.3 ± 4.6 ^a^	44.5 ± 6.1 ^b^	57.3 ± 5.8 ^b^

Different superscript letters indicate significant differences at *p* < 0.05.

**Table 6:** Evolution of in vitro inhibition of vesperlysine- and pentosidine-like advanced glycation end products (AGE) before (uncooked), and after traditional cooking (cooked) and after traditional cooking followed by in vitro gastro-intestinal digestion (in vitro Digestion) from beans from ten Fabaceae species.

Plant Species	Vesperlysine-Like AGEs (Inhibition %)			Pentosidine-Like AGEs (Inhibition %)		
	Uncooked	Cooked	In Vitro Digestion	Uncooked	Cooked	In Vitro Digestion
*P. sativum*	47.4 ± 5.8 ^a^	37.4 ± 4.6 ^a^	44.7 ± 5.7 ^a^	31.9 ± 3.3 ^a^	17.1 ± 1.4 ^b^	20.5 ± 2.4 ^b^
*C. cajan*	26.4 ± 4.8 ^a^	21.0 ± 4.1 ^a^	24.1 ± 2.3 ^a^	20.0 ± 1.9 ^a^	16.2 ± 2.8 ^a^	17.8 ± 3.1 ^a^
*V. unguiculata*	35.3 ± 1.5 ^a^	22.4 ± 3.9 ^b^	26.9 ± 3.8 ^ab^	35.3 ± 2.8 ^a^	15.3 ± 3.4 ^b^	14.3 ± 1.6 ^b^
*V. mungo*	49.1 ± 6.9 ^a^	23.1 ± 4.5 ^c^	41.2 ± 4.2 ^b^	47.5 ± 2.9 ^a^	19.0 ± 3.1 ^b^	26.3 ± 1.9 ^b^
*P. vulgaris*	46.2 ± 1.8 ^b^	26.5 ± 3.3 ^c^	52.7 ± 6.1 ^a^	42.6 ± 3.9 ^a^	18.7 ± 2.9 ^c^	35.2 ± 2.2 ^b^
*G. max*	47.3 ± 5.2 ^a^	31.2 ± 2.8 ^b^	47.5 ± 3.7 ^a^	34.2 ± 2.3 ^b^	15.4 ± 4.3 ^c^	56.0 ± 3.8 ^a^
*V. angularis*	49.9 ± 2.6 ^a^	33.3 ± 1.7 ^b^	35.5 ± 8.5 ^b^	51.2 ± 3.7 ^a^	23.8 ± 3.1 ^b^	22.4 ± 2.9 ^b^
*A. hypogaea*	48.2 ± 3.1 ^a^	21.8 ± 3.1 ^c^	30.4 ± 5.2 ^b^	27.2 ± 3.4 ^a^	16.2 ± 2.6 ^b^	22.1 ± 2.5 ^ab^
*V. radiata*	41.1 ± 2.0 ^a^	20.3 ± 4.3 ^c^	30.1 ± 3.5 ^b^	24.1 ± 3.7 ^a^	15.8 ± 4.6 ^ab^	15.7 ± 1.5 ^b^
*V. unguiculata* subsp. *sesquipedalis*	50.6 ± 7.1 ^a^	36.6 ± 1.6 ^b^	40.6 ± 3.9 ^b^	50.8 ± 6.8 ^a^	21.4 ± 2.6 ^c^	29.7 ± 1.7 ^b^

Different superscript letters indicate significant differences at *p* < 0.05.

**Table 7:** Evolution of in vitro inhibition of α-amylase and α-glucosidase enzymes, and cellular glucose uptake by yeast cells before (uncooked), and after traditional cooking (cooked) and after traditional cooking followed by in vitro gastro-intestinal digestion (in vitro Digestion) from beans from ten Fabaceae species.

Plant Species	α-Amylase (% Inhibition)			α-Glucosidase (% Inhibition)			Glucose Uptake (% Increase)		
	Uncooked	Cooked	In Vitro Digestion	Uncooked	Cooked	In Vitro Digestion	Uncooked	Cooked	In Vitro Digestion
*P. sativum*	41.6 ± 1.4 ^a^	24.7 ± 2.0 ^c^	35.4 ± 1.9 ^b^	30.9 ± 2.3 ^a^	24.5 ± 1.8 ^b^	33.0 ± 1.3 ^a^	32.8 ± 1.5 ^a^	27.7 ± 3.8 ^a^	31.3 ± 3.1 ^a^
*C. cajan*	24.7 ± 3.4 ^a^	16.8 ± 1.4 ^b^	17.7 ± 2.6 ^ab^	21.0 ± 2.2 ^a^	17.3 ± 2.4 ^a^	18.7 ± 1.5 ^a^	27.9 ± 1.7 ^a^	24.1 ± 2.6 ^ab^	24.2 ± 1.4 ^b^
*V. unguiculata*	38.1 ± 3.4 ^a^	15.6 ± 1.3 ^b^	18.1 ± 1.9 ^b^	29.3 ± 1.3 ^a^	19.4 ± 1.3 ^b^	19.4 ± 0.7 ^b^	30.1 ± 2.4 ^a^	23.6 ± 2.9 ^b^	25.1 ± 3.1 ^ab^
*V. mungo*	47.8 ± 2.8 ^a^	17.4 ± 0.9 ^c^	38.6 ± 2.3 ^b^	42.6 ± 3.3 ^a^	18.9 ± 2.1 ^c^	33.2 ± 1.8 ^b^	44.6 ± 1.8 ^a^	25.2 ± 3.3 ^c^	33.4 ± 1.4 ^b^
*P. vulgaris*	32.5 ± 2.3 ^a^	16.2 ± 2.8 ^b^	27.6 ± 2.5 ^a^	31.1 ± 2.5 ^b^	20.4 ± 1.8 ^c^	37.0 ± 1.4 ^a^	39.5 ± 1.2 ^b^	26.8 ± 2.8 ^c^	51.3 ± 2.4 ^a^
*G. max*	29.2 ± 2.4 ^a^	15.1 ± 0.6 ^b^	28.7 ± 1.6 ^a^	25.3 ± 2.4 ^b^	20.8 ± 0.9 ^c^	40.6 ± 3.6 ^a^	32.2 ± 2.0 ^b^	32.8 ± 1.8 ^b^	55.3 ± 4.6 ^a^
*V. angularis*	50.8 ± 2.7 ^a^	24.6 ± 1.4 ^c^	39.3 ± 1.9 ^b^	44.5 ± 3.6 ^a^	25.2 ± 1.5 ^c^	34.2 ± 1.4 ^b^	48.4 ± 1.7 ^a^	30.7 ± 3.2 ^b^	33.6 ± 1.7 ^b^
*A. hypogaea*	34.5 ± 2.6 ^a^	15.6 ± 1.5 ^c^	22.2 ± 2.6 ^b^	32.8 ± 1.2 ^a^	16.3 ± 0.9 ^c^	25.6 ± 0.7 ^b^	36.2 ± 2.4 ^a^	21.8 ± 1.9 ^b^	34.3 ± 3.2 ^a^
*V. radiata*	28.2 ± 1.7 ^a^	16.2 ± 2.3 ^b^	15.5 ± 1.7 ^b^	24.7 ± 2.6 ^a^	16.5 ± 1.4 ^b^	23.3 ± 3.2 ^a^	28.1 ± 1.9 ^b^	21.5 ± 3.2 ^c^	35.4 ± 1.7 ^a^
*V. unguiculata* subsp. *sesquipedalis*	58.7 ± 3.8 ^a^	25.2 ± 1.5 ^c^	38.8 ± 1.9 ^b^	44.6 ± 4.5 ^a^	26.8 ± 1.8 ^c^	35.0 ± 1.7 ^b^	43.9 ± 1.2 ^a^	31.2 ± 1.8 ^b^	36.2 ± 2.5 ^b^

Different superscript letters indicate significant differences at *p* < 0.05.

## Data Availability

All the data supporting the findings of this study are included in this article.

## Supplementary Materials

The following are available online at https://www.mdpi.com/article/10.3390/plants11010067/s1, Table S1. The collected 10 taxa of the Fabaceae beans species cover the whole floristic regions in Thailand; Table S2. Pearson correlation coefficient linking phytochemicals antioxidant and anti-diabetic activities of extracts from beans from ten Fabaceae species subjected to traditional cooking and in vitro gastrointestinal digestion.

## Sample Availability

Samples presented in this study are available from the authors upon request.
