# Supplementary material for: Effect of Traditional Cooking and In Vitro Gastrointestinal Digestion of the Ten Most Consumed Beans from the Fabaceae Family in Thailand on Their Phytochemicals, Antioxidant and Anti-Diabetic Potentials
Source: Plants (Basel). 2021 Dec 26;11(1):67. doi: 10.3390/plants11010067 (PMC8747412; doi:10.3390/plants11010067)
Supplement: Supplementary file 1 [file plants-11-00067-s001.zip › plants-1495124-supplementary.pdf]

Supplementary Materials

Effect of Traditional Cooking and *In Vitro* Gastrointestinal Digestion of the Ten Most Consumed Beans from the Fabaceae Family in Thailand on Their Phytochemicals, Antioxidant and Anti-Diabetic Potentials

Duangjai Tungmunnithum [1,2,3,*], Samantha Drouet [2], Jose Manuel Lorenzo [4,5] and Christophe Hano [2,3,*]

1 Department of Pharmaceutical Botany, Faculty of Pharmacy, Mahidol University, Bangkok 10400, Thailand
2 Laboratoire de Biologie des Ligneux et des Grandes Cultures, INRAE USC1328, Campus Eure et Loir, Orleans University, 28000 Chartres, France; samantha.drouet@univ-orleans.fr
3 Le Studium Institue for Advanced Studies, 1 Rue Dupanloup, 45000 Orleans, France
4 Centro Tecnológico de la Carne de Galicia, Adva. Galicia n° 4, Parque Tecnológico de Galicia, San Cibrao das Viñas, 32900 Ourense, Spain; jmlorenzo@ceteca.net
5 Área de Tecnología de los Alimentos, Facultad de Ciencias de Ourense, Universidad de Vigo, 32004 Ourense, Spain
* Correspondence: duangjai.tun@mahidol.ac.th (D.T.) ; hano@univ-orleans.fr (C.H.)

Table S1. The collected 10 taxa of the Fabaceae beans species cover the whole floristic regions in Thailand.

Table S2: Pearson correlation coefficient linking phytochemicals antioxidant and anti-diabetic activities of extracts from beans from ten Fabaceae species subjected to traditional cooking and in vitro gastrointestinal digestion.

Table S1. The collected 10 taxa of the Fabaceae beans species cover the whole floristic regions in Thailand.

Taxon No.	Scientific Name	Floristic regions	Locallities
1	*Pisum sativum*	N	Lampang
		PEN	Surat Thani
2	*Cajanus cajan*	SW	Chumphon
		E	Yasothon
3	*Vigna unguiculata*	C	Chainat
4	*Vigna unguiculata* subsp. *sesquipedalis*	N	Lampang
		N	Chiang Mai
		N	Nakhon Sawan
		E	Chaiyaphum
5	*Vigna radiata*	N	Lamphun
		N	Lampang
		N	Chiang Mai
		N	Nakhon Sawan
		E	Buriram
6	*Vigna mungo*	E	Ubon Ratchathani
		N	Sukhothai
		NE	Nakhon Phanom
		E	Ubon Ratchathani
		N	Phichit
		SW	Uthai Thani
7	*Vigna angularis*	C	Lop Buri
		SW	Kanchanaburi
		SW	Phetchaburi
		SE	Chanthaburi
		SE	Prachin Buri
		C	Ang Thong
8	*Phaseolus vulgaris*	NE	Phetchabun
		SE	Prachin Buri
		N	Phichit
		N	Phitsanulok
		C	Chainat
		N	Chiang Mai
		N	Chiang Rai
		N	Mae Hong Son
		SE	Trat
		N	Tak
		SW	Uthai Thani
		C	Suphan Buri
		C	Pathum Thani
9	*Glycine max*	N	Chiang Mai
		NE	Kalasin
		NE	Loei
		N	Uttaradit
		N	Kamphaeng Phet
		N	Nan
10	*Arachis hypogaea*	N	Nan

NE	Phetchabun
N	Phayao
E	Surin
C	Nakhon Nayok

Table S2: Pearson correlation coefficient linking phytochemicals antioxidant and anti-diabetic activities of extracts from beans from ten Fabaceae species subjected to traditional cooking and *in vitro* gastrointestinal digestion.

	TPC	TFC	TAC	DPPH	ABTS	FRAP	CAA	vespAGE	pentAGE	AMYL	GLUC
**TPC**											
**TFC**	0.730 ***										
**TAC**	0.464 **	0.775 **									
**DPPH**	0.552 **	0.596 **	0.627 ***								
**ABTS**	0.463 *	0.598 ***	0.719 ***	0.888 ***							
**FRAP**	0.766 ***	0.747 ***	0.572 ***	0.640 ***	0.618 ***						
**CAA**	0.638 ***	0.545 **	0.467 ***	0.506 ***	0.537 **	0.769 ***					
**vespAGE**	0.847 ***	0.591 ***	0.333 ns	0.432 *	0.354 ns	0.699 ***	0.751 ***				
**pentAGE**	0.691 ***	0.731 ***	0.536 *	0.452 *	0.478 ***	0.645 ***	0.718 ***	0.788 ***			
**AMYL**	0.829 ***	0.863***	0.611 ***	0.646 ***	0.580 **	0.897 ***	0.790 ***	0.789 ***	0.768 ***		
**GLUC**	0.855 ***	0.808 ***	0.477 **	0.476 ***	0.418 *	0.784 ***	0.686 ***	0.880 ***	0.863 **	0.8928 **	
**Glc uptake**	0.689 ***	0.581 ***	0.265 ns	0.256 ns	0.237 ns	0.495 ns	0.530 *	0.809 *	0.848 **	0.605 **	0.876 ***

*** significant $p < 0.001$; ** significant $p < 0.01$; * significant $p < 0.05$; ns: non-significant $p > 0.05$. TPC: total phenolic content; TFC: total flavonoid content; MAC: monomeric anthocyanin content; DPPH: in vitro antioxidant DPPH assay; ABTS: in vitro antioxidant ABTS assay; FRAP: in vitro antioxidant FRAP assay; CAA: cellular antioxidant assay; vespAGE: inhibition of vesperlysine-like AGEs formation; pentAGE: inhibition of pentosidine-like AGEs formation; AMYL: inhibition of α-amylase acitivity; GLUC: inhibition of α-glucosidase activity; Glc uptake: increase of cellular glucose uptake.
